# Mandibular device treatment in obstructive sleep apnea -A structured therapy adjustment considering night-to-night variability night-to-night variability in mandibular devices

**DOI:** 10.1007/s11325-024-03134-w

**Published:** 2024-09-06

**Authors:** Greta Sophie Papenfuß, Inke R. König, Christina Hagen, Alex Frydrychowicz, Fenja Zell, Alina Janna Ibbeken, Thorsten M. Buzug, Ulrike Kirstein, Lina Kreft, Daniel Grünberg, Samer Hakim, Armin Steffen

**Affiliations:** 1 Department of Otorhinolaryngology, University of Lübeck, Campus Lübeck, Ratzeburger Allee 160, 23538 Lübeck, Germany; 2 Institute of Medical Biometry and Statistics, University of Lübeck, Lübeck, Germany; 3 Fraunhofer Research Institution for Individualized and Cell-Based Medical Engineering IMTE, Lübeck, Germany; 4 Department of Radiology, University of Schleswig-Holstein, Campus Lübeck, Germany; 5 Institute of Medical Engineering, University of Lübeck, Lübeck, Germany; 6 HICAT GmbH, SICAT GmbH & Co. KG, Bonn, Germany; 7 Department of Oral and Maxillofacial Surgery, University of Lübeck, Lübeck, Germany; 8 Department of Oral and Maxillofacial Surgery, Helios Medical Centre, Schwerin, Germany

**Keywords:** Obstructive sleep apnea, Mandibular advancement device, Night-to-night variability, Patient related outcome

## Abstract

**Background:**

Mandibular advancement devices (MAD) are a well-established treatment option for obstructive sleep apnea (OSA). MAD are considered preferably for patients with mild to moderate OSA presenting with a elevated night-to-night variability (NNV). This study aimed to determine the treatment effect of MAD on NNV considering different protrusion distances and patient related outcome (PRO).

**Methods:**

We conducted a prospective cohort analysis of patients before MAD with 60% and 80% of the maximum protrusion. OSA severity was assessed using a home-sleep test for two consecutive nights. PRO contained the Epworth Sleepiness Scale (ESS) and sleep related quality of life (FOSQ).

**Results:**

Twenty patients with a median overweight body-mass-index of 27.1 (interquartile range (IQR) 16.3 kg/m²), with a mainly mild to moderate OSA with an apnea -hypopnea index (AHI) of 18.3 / h (IQR 17.7) and elevated ESS of 12.5 (IQR 8.0) were included. As opposed to 80%, 60% protrusion significantly but not 80% relevantly reduced AHI (60%%: 11.2 (IQR 5.5)/h, *p* = 0.01; 80%: 12.9 (IQR18,0)/h, *p* = 0.32) and improved the ESS (60%: 8.0 (IQR 10,0); 80%: 10 (IQR 9.0)), with therapy settings. No correlation could be detected between NNV and ESS, and FOSQ changes. Higher baseline NNV was associated with severe OSA (*p* = 0.02) but not with gender, overweight, or status post-tonsillectomy.

**Conclusions:**

OSA improvement is associated with lower NNV; both OSA and NNV are connected to the degree of protrusion. Therefore, higher NNV does not justify the exclusion of candidates for MAD treatment. PRO changes are not visibly affected by NNV but by general OSA changes. These findings may help to define and optimize future study designs for the primary outcome decision between objective OSA parameters and PRO.

## Introduction

Obstructive sleep apnea (OSA) is a worldwide and common sleep related breathing disorder [1]. Mild and moderate OSA as defined by an apnea- hypopnea index (AHI) below 30/h account for the vast majority of cases [2]. Mandibular advancement devices (MAD) are considered to be an effective treatment option to treat excessive daytime sleepiness and several other psychometric dimensions as evidenced elsewhere, especially in those patients with mild or moderate OSA [3]. Over the last years, OSA research and health care decision makers increasingly focused on patient related outcome by adding the individual patient’s perspective to routine clinical parameters such as AHI and oxygen desaturation index. These technically driven parameters showed to be prone to misinterpretation, e.g., due to night-to-night variability. Night-to-night variability is most common in mild to moderate OSA [4, 5], can lead to misdiagnosing OSA, and may negatively affect the assessment of treatment effects. Hence, night-to-night variability warrants increased attention in patients with mild to moderate OSA.

Therefore, we aimed to assess the treatment effect of MAD on night-to-night variability considering different protrusion levels and patient related outcome taking into account that an increased night-to-night variability may have negative impact on patient benefit. Patient related outcome included (i) the Epworth Sleepiness Scale (ESS) as a validated psychometric instrument for daytime sleepiness and (ii) the Functional Outcome of Sleep Questionnaire (FOSQ) as an indicator for sleep related quality of life.

## Patients and methods

### Study design, devices and sleep assessment

We conducted a prospective cohort study of patients before MAD. MAD with 60% and 80% of the maximum protrusion were applied in patients of the I-SLEEP study cohort. This study focused on combining magnet resonance imaging of the upper airway and different degrees of mandibular protrusion in OSA (BMBF Grant Number 13GW0276B). In this research project, the industrial partner provided the MAD for all participants (Optisleep/HICAT GmbH, Bonn).

**Fig. 1:**
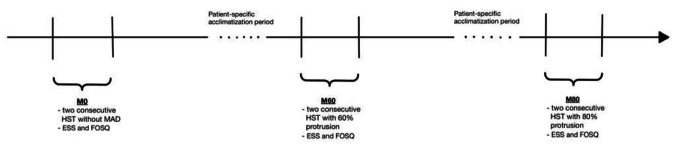
Study design detailing the evaluation time points of the study. FOSQ – Functional Outcome of Sleep Questionnaire, ESS – Epworth Sleepiness Scale, HST – Home Sleep Test, MAD - Mandibular Advancement Device

OSA severity was assessed using a home-sleep test (HST) for two consecutive nights at baseline (M0) and at two follow-up time-points after MAD application with 60% (M60) and with 80% (M80) of maximum protrusion as depicted in Fig. 1. To determine each patients’ mandibular protrusion capacity a bite fork was used: Starting at 0° (neutral), the patient were to advance the mandible as far forward as possible (100% protrusion). Partial protrusion (60% and 80%, respectively) were determined from each individuals’ maximum. For inclusion in the study, at least four out of six scheduled HST needed to be successfully completed. Missing values were marked in the respective analyses.

For HST, we used peripheral arterial tonometry (WatchPAT ^®^/Itamar) at baseline and throughout follow-up in order to reduce the potential risk of interrater variability and inaccuracy as the same method was used. Besides having an AHI above 5 events per hour there were no restrictions regarding OSA severity, or, overweight expressed by elevated body mass index (BMI). Patient were selected after dental consulting regarding appropriate temporomandibular joint function and dental status. Adult patients were admitted to the study irrespective of prior positive airway pressure therapy or being therapy naïve.

Participants were asked to fill in the Epworth Sleepiness Scale (ESS) [6] for the assessment of daytime sleepiness and the Function Outcomes Of Sleep questionnaire (FOSQ) [7] for the evaluation of sleep related quality of life at baseline and each of the two follow-up time points M60 and M80, respectively. Both tests are self-administered psychometric tests with normative values but with opposing modes of interpretation: Higher ESS values (≥ 11) are associated with higher daytime sleepiness [8], higher FOSQ values (≥ 17.9) are regarded to express a higher sleep related quality of life [7].

### Ethics approval and research funding

Local ethic committee approval was granted prior to inclusion of participants who gave written consent (AZ 19–021). The authors acknowledge funding by the German federal ministry of Education and Research (BMBF Grant Number 13GW0276B).

### Statistical analysis

We used Jamovi Version 2.0.0 for all analyses. Descriptive statistics were calculated for the demographic variables. The results are given as the median and interquartile range if not labelled separately. Continuous variables (HST between two consecutive nights, between baseline and each of the two follow-up time-points at 60% (M60) and 80% (M80) protrusion) were compared using Mann-Whitney-U test and Friedman test (repeated measures ANOVA). P values ≤ 0.05 were considered statistically relevant but without claiming significance. The analyses are regarded as being descriptive, so final conclusions need to be assured.

The results were also analyzed graphically by using box plots, Bland-Altman plots and Q-Q plots. Q-Q plots were used to check data graphically for normal distribution. Bland-Altman plots were applied to analyze the variability of measurements of two consecutive nights in terms of night-to-night variability. Correlation analysis of FOSQ, ESS, and night-to-night variability (AHI differences between the nights) was achieved using Spearman’s correlation coefficient.

## Results

### Cohort description

**Fig. 2:**
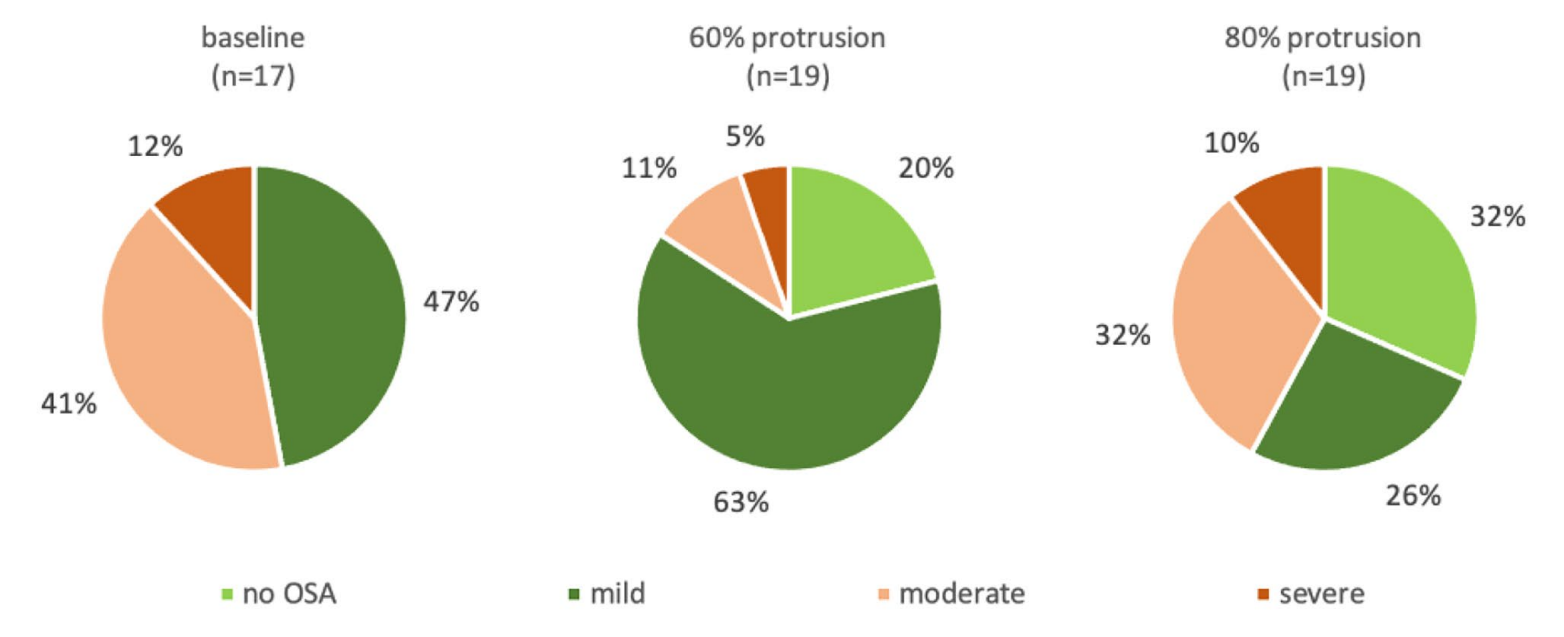
Distribution of OSA severity at baseline and during therapy at MAD with 60% and 80% protrusion, respectively. Note that at baseline, data from three participants and at M60 and M80 data from one participant was missing

There were 20 participants fulfilling the selection criteria (Table 1). The cohort was almost gender balanced, middle-aged with a median 49 years (IQR 16.3) and revealed a median overweight BMI of 27.1 kg/m² (IQR 4.7). A mainly mild to moderate OSA (Fig. 2), elevated ESS of 12.5 points (IQR 8.0), and a decreased sleep related quality of life according to FOSQ of 13.6 points (IQR 6.3). 80% of participants had previous experience with positive airway pressure therapy, 20% of participants sustained a tonsillectomy for OSA and other indications. At baseline, one third of patients revealed an at least two-fold increased AHI in supine position as compared to non-supine, and half of the candidates suffered REM-dependent OSA with an at least two-fold increased REM-AHI compared to non-REM sleep.

### Home sleep test and patient related outcome analysis

As detailed in Table 1, several parameters such as AHI, oxygen desaturation index, or time ratio below 90% oxygen desaturation improved at 60% protrusion. Further increase to 80% protrusion diminished the effect in relation to 60% protrusion but not to baseline (Table 1). The ratio of patients having mild or no OSA increased with therapy but was lower at 80% protrusion as compared to 60% protrusion (Fig. 2). There was no statistically relevant change in AHI specific for REM or supine sleep. Next to the increased ratio of sleep in supine position during MAD as compared to baseline (Table 1), there was no relevant change between the 60% and 80% MAD. However, there was a small but statistically relevant change of the time portion with REM sleep between MAD at 60% and 80%. ESS and FOSQ values improved between baseline and MAD. However, changes reached statistical relevance comparing baseline and MAD at 80% protrusion. At baseline, high ESS values were reported in 65% of the patients. During MAD with 60% and 80% protrusion the ratio decreased to 35% and 40%, respectively 70% of the participants showed lower FOSQ values hinting at decreased sleep related quality of life. FOSQ values improved to 40% and 50% with 60% and 80% protrusion, respectively.

**Table 1:** Home sleep test and patient related outcome analysis at baseline and with therapy. FOSQ – functional outcome of Sleep Questionnaire, ESS – Epworth Sleepiness Scale, AHI – apnea-hypopnea index, REM – rapid eye movement, ODI- oxygen desaturation index, SaO2 – oxygen saturation, IQR- interquartile range; p values with Friedman test

	Baseline (median (IQR))	60% protrusion (median (IQR))	80% protrusion (median (IQR))	*p* value Baseline to 60% protrusion	*p* value Baseline to 80% protrusion	*p* value 60–80% protrusion
**FOSQ** (in points)	13.6 (6.3) (*n* = 19)	18.2 (4.5) (*n* = 18)	16.6 (6.6) (*n* = 18)	0.09	**0.029**	1
**ESS** (in points)	12.5 (8.0) (*n* = 20)	8.0 (10.0) (*n* = 18)	10 (9.0) (*n* = 17)	0.197	**0.008**	0.564
**AHI** (in events per hour)	18.3 (17.7) (*n* = 17)	11.2 (5.5) (*n* = 19)	12.9 (18.0) (*n* = 19)	**0.012**	0.317	0.637
**REM associated AHI** (in events per hour)	23.4 (20.0) (*n* = 20)	20.4 (17.7) (*n* = 18)	17.7 (24.8) (*n* = 18)	0.593	0.052	0.593
**Supine AHI** (in events per hour)	24.9 (17.8) (*n* = 18)	15.7 (9.0) (*n* = 18)	17.9 (20.5) (*n* = 19)	0.096	0.564	0.796
**ODI** (in events per hour)	6.4 (11.9) (*n* = 17)	3.7 (2.75) (*n* = 19)	3.3 (6.7) (*n* = 19)	**0.012**	**0.046**	0.617
**Minimal SaO2** (in %)	87 (6.5) (*n* = 20)	89.5 (5.8) (*n* = 19)	88.5 (4.3) (*n* = 19)	0.197	**0.046**	1
**Time below 90% SaO2** (in %)	0.3 (0.9) (*n* = 20)	0 (0.4) (*n* = 19)	0.1 (0.3) (*n* = 19)	**0.021**	0.058	0.317
**Ratio of REM sleep** (in %)	29.4 (3.2) (*n* = 20)	29.3 (7.8) (*n* = 18)	25.7 (8.1) (*n* = 18)	0.285	0.071	**0.046**
**Ratio of sleep in supine position** (in %)	39.3 (24.0) (*n* = 19)	50.4 (35.8) (*n* = 18)	50.8 (34.0) (*n* = 19)	0.197	**0.029**	0.796

### Night-to-night variability (NNV)

**Fig. 3:**
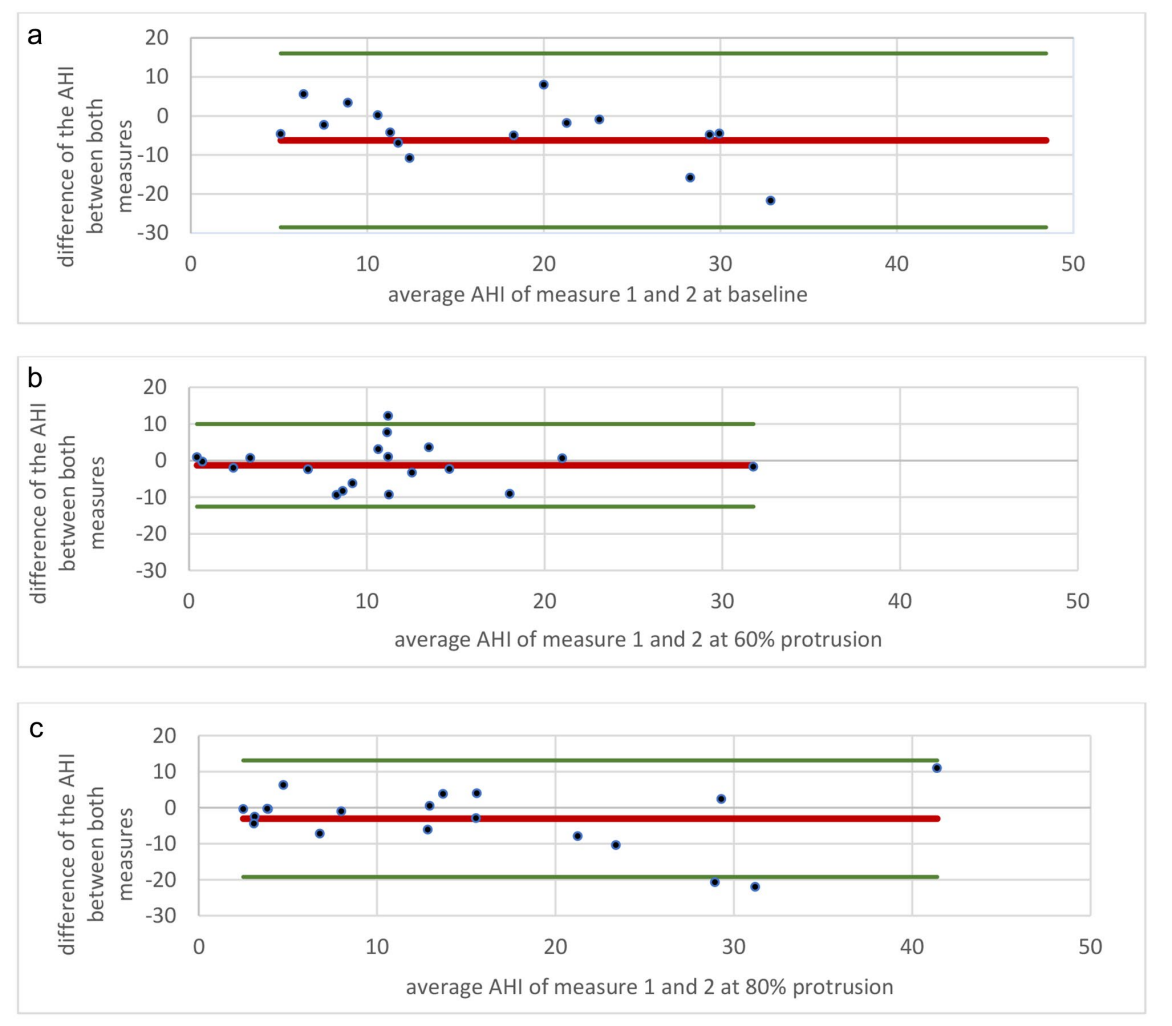
**a** Bland Altman plot for baseline apnea-hypopnea index (AHI) measures. **b**: Bland Altman plot apnea-hypopnea index (AHI) measures at 60% protrusión. **c**: Bland Altman plot for apnea-hypopnea index (AHI) measures at 80% protrusion

NNV as expressed by AHI differences was higher between baseline HSTs as compared with 60% and 80% MAD HSTs. Bland-Altman-windows of baseline HSTs, 60% MAD and 80% MAD HSTs show that 60% MAD showed a higher decrease of NNV (Fig. 3a-c).The Bland-Altman diagram shows the relationship between the mean of two values and their difference. By calculating the mean and standard deviation of these differences, we can determine the confidence limits. We plotted the mean AHI values from the first and second night of the baseline, the 60% measurement, and the 80% measurement against their differences. The 95% confidence intervals were largest for the baseline, smallest for the 60% measurement, and medium range for the 80% measurement. This indicates a large deviation at baseline, which significantly decreases under 60% therapy and increases slightly under 80% therapy, but remains reduced compared to baseline. Also, in REM-AHI variation was higher at baseline than during therapy, it decreases continuously and was therefore lowest at 80% protrusion. For the AHI in supine position, the observation was the opposite, there was more variation with therapy.

Low FOSQ values indicating decreased sleep related quality of life correlated with higher daytime sleepiness as higher ESS values at baseline and both protrusion settings (*p* < 0.001 each). There was no statistically relevant correlation between night-to-night variability and ESS. FOSQ changes besides the single association that participants with non-suspicious high ESS had relevant more night-to-night variability with 80% protrusion (*p* = 0.036).

In order to explore potential baseline parameters for predicting night-to-night variability, only severe but not mild or moderate OSA (*p* = 0.02) was associated with higher baseline night-to-night variability. There was no statistically relevant correlation between night-to-night variability to either gender, overweight expressed by body mass index, status post tonsillectomy, or previous positive airway pressure therapy.

## Discussion

This is one of few studies successfully analyzing the effect of MAD treatment and its impact on night-to-night variability of the AHI and patient reported outcome over the time course of a typical therapy adjustment pathway. In our cohort night-to-night variability of the AHI was attenuated by therapy and appears to have no correlation with patient related outcome and other parameters such as overweight or gender. Patients with severe OSA had higher night-to-night variability.

MAD is a well-established treatment for OSA with documented effects on various aspects of patient related outcome such as daytime sleepiness reduction and quality of life [3, 9]. Nonetheless, there are reports about predictive parameters that negatively affect treatment response or might negatively impact ongoing MAD therapy such as overweight or weight gain [10, 11], severe OSA [12], or insomnia [13]. Our study addressed the question whether higher night-to-night variability of OSA might have negative impact on treatment effects. Night-to-night variability is a phenomenon in OSA that increases with changing patient´s habits such as alcohol intake [14, 15] and is reported to be more relevant in mild to moderate OSA [16–19]. In mild to moderate OSA, higher rates of patients with positional OSA are documented [20, 21]. As non-severe and positional OSA have previously been described as positive predictors for MAD response, these aspects were closely analyzed in our study. To our surprise, except for severe OSA there were no associations between night-to-night variability, OSA parameters, and patient related outcome. Instead, treatment with MAD seemed to reduce night-to-night variability itself (Fig. 3a-c). Patient related outcome appears to be less sensitive to night-to-night variability, i.e., alternating nights with effective and less effective reduction of OSA.

In line with the mechanism of action of the mandibular advancement splint as a means of preventing the tongue from falling back, the AHI in supine position decreased under therapy, while the proportion of sleep in the supine position and the sleep time in the supine position increased (Table 1). A possible explanation may be that the supine position during sleep is considered “safer” again and as a results will no longer be subconsciously prevented as often, as indicated by fewer apnea and hypopnea events during these phases. To our astonishment, MAD therapy affected the entire night´s AHI more effectively than the REM-AHI or AHI in a supine position which indicates that dominantly tongue base obstruction appeared to have higher response rates [22]. On the other hand, our findings are in line with other response assessment in large cohorts [12]. As our cohort was established with only minimal exclusion criteria and covers a broad variety of overweight, gender, and OSA degree, it is quite unclear where this result arises from and is subject to larger patient cohorts.

Surprisingly, we found that increased mandibular protrusion did not reduce the AHI; instead, AHI slightly increased. This indicates an overshooting therapeutic and a dose-dependent effect. Excessive mandibular advancement may have counterproductive effects, such as temporomandibular joint discomfort, leading to reduced sleep quality. Determining the optimal percentage of mandibular advancement for each patient, minimizing side effects, requires individual titration within a specific range.

Our data include several potential shortcomings. First, this case series included a limited number of patients without power calculation. The reason for this is the initial proof of concept approach of the main underlying I-SLEEP study focusing on changes of upper airway dimensions. The proof-of-concept approach was also the reason why no randomization was performed. The unexpected finding that a 60% protrusion led to greater improvement than an 80% protrusion, was serendipitous. With our data and the findings of a dose related effect, effects could be estimated in a randomized setting and group sizes could be planned accordingly. Future studies with randomization could yield further interesting insights. Second, the results indicate that increasing to 80% reduces the outcome as compared to 60%. However, it cannot be definitively ruled out that this trend is related to the study design. Especially for patient related outcomes different effects may become apparent in a randomized crossover trial not limited to 60% protrusion first and 80% protrusion last. However, in light of results in previous randomized crossover studies with one week interval [23] we consider this effect to be of lesser importance. Third, we relied on HST based on peripheral artery tonometry rather than polysomnography in order to minimize interrater variability. What may be seen as a shortcoming is a strength of our data as results represent real-life data [24] and could fulfill simpler hygiene requirements during the COVID pandemic. Lastly, there are MAD applications available today that assess usage, i.e., therapy adherence or, even more interesting for evaluating night-to-night variability, calculate a respiratory disturbance index per nightly utilization of the MAD [25]. These concepts were too experimental at the beginning of our study and not commonly available in sleep medicine. Nowadays, they offer new opportunities for parametric insights of night-to-night variability in MAD therapy. Ultimately, we observed a consistent distribution of missing values and measurements across the three measurement time points, with no significant loss of participants over the course of the therapy. There were no notable dropouts or increased absences based on the level of protrusion.

In conclusion, in our sample OSA improvement is associated with lower night-to-night variability; both are connected to the degree of protrusion. Patient related outcome changes are not visibly affected by night-to-night variability but by general OSA changes. Therefore, higher night-to-night variability does not justify to exclude candidates for MAD treatment. These findings may help to define future study designs for the primary outcome decision between objective OSA parameters and patient related outcome.

## Data Availability

Our manuscript has no associated data.
